# Why is dimerization essential for class-C GPCR function? New insights from mGluR1 crystal structure analysis

**DOI:** 10.1007/s13238-014-0062-z

**Published:** 2014-05-09

**Authors:** Xuejun C. Zhang, Jianfeng Liu, Daohua Jiang

**Affiliations:** 1 National Laboratory of Macromolecules, National Center of Protein Science—Beijing, Institute of Biophysics, Chinese Academy of Sciences, Beijing, 100101 China; 2 School of Life Science and Technology, Huazhong University of Science and Technology, Wuhan, 430074 China

G-protein coupled receptors (GPCRs) play an essential role in eukaryotic cells signaling. According to phylogenetic analysis, most GPCRs belong to one of four classes, i.e. A, B, C, and Frizzled (Lagerstrom and Schioth, 2008). The class-C GPCR family contains metabotropic glutamate receptors (mGluR), γ-aminobutyric acid B receptors (GABA_B_ receptor or GBR), several taste-sensing receptors (e.g. TAS1), and a Ca^2+^-sensing receptor (CaS). One characteristic feature of the class-C GPCRs is their dimerization, either into homo- or hetero-dimers, which is obligatory for function (Kniazeff et al., 2011). Each subunit in the dimer usually consists of an amino (N)-terminal extracellular domain (ECD) which binds orthosteric agonists, a linker peptide, a 7-transmembrane (TM) domain, and a cytosolic tail of varying lengths at the carboxyl (C)-terminus. It is well-established that all domains spare the linker peptide participating in the dimerization process (Rondard et al., 2008; Huang et al., 2011; Rondard et al., 2006). Further, it is believed that, after transmission of the ligand-binding signal from the ECD region to the 7-TM domain, the activation of the class-C GPCR dimer is associated with a conformational change of the 7-TM domain on the cytosol side, enabling binding and activation of downstream G-proteins. In contrast, the precise details of the mechanisms for signal transduction from the ligand binding site in the ECD region to the cytosolic side of the 7-TM domain, where downstream G-proteins are activated, remain to be elucidated.

Recently, the crystal structure of mGluR1 (including the linker peptide and the 7-TM domain), the first one from the class-C GPCRs, has been reported at 2.8-Å resolution (PDB ID: 4OR2) (Wu et al., 2014). Now, three-dimensional structures of all four classes are elucidated (Okada et al., 2000; Cherezov et al., 2007; Rasmussen et al., 2011; Hollenstein et al., 2013; Wang et al., 2013). The following observations were made from the mGluR1 crystal structure: (1) mGluR1 exists as a symmetrical homodimer. The dimer interface is formed by the TM helices 1 and 2 from both subunits, and there is a cluster of six cholesterol molecules located in the extracellular half of the interface. In order to maintain the interaction with the cholesterol cluster, each mGluR1 subunit places its Trp588 in a particular rotamor that requires position 650 of the same subunit to be a small side chain residue, e.g. Ala. These two positions are highly conserved in most class-C GPCRs, and they co-evolve to Leu and/or Ile residues in GBR, suggesting that the dimerization interface observed in the mGluR1 crystal structure is conserved among other class-C GPCRs as well. (2) One molecule of a negative allosteric modulator (NAM), FITM, was found to bind in a pocket on the extracellular side of the 7-TM domain, which is in a similar location of binding sites of orthosteric ligands in class-A GPCRs. Therefore, in the presence of a negative allosteric modulator, both subunits assume an inactive conformation. (3) In this inactive conformation, multiple interactions were observed between the linker peptide and extracellular loop 2 (ECL2) of the 7-TM domain. (4) Between TMs 3 and 6 exists an ionic lock, similar to that conserved in class-A GPCRs. These structural observations strongly suggest that the 7-TM domain of mGluR1 (and happens of other class-C GPCRs) shares a number of common activation mechanisms with the 7-TM domains of class-A GPCRs. In particular, signal transduction through the 7-TM is likely to be initiated by a local conformational change on the extracellular side, probably near the observed NAM-binding pocket. According to structures of active class-A GPCRs (Rasmussen et al., 2011), activation of the 7-TM domain by binding of an agonist on the extracellular side is through reorganization of the packing of the 7 TM helices and results in a large conformational change on the cytosolic side, enabling G-protein binding (Zhang et al., 2013). The question on class-C GPCRs remains, however, as to how the ligand-binding signal is transferred from the dimerized ECDs to the dimerized 7-TM.

In all probability, there are two general ways in which the ECD can activate the 7-TM domain upon agonist binding: (1) by providing mechanical energy to induce the conformational change in the 7-TM domain, and/or (2) by generating a neo-ligand that triggers the conformational change. Although the ECD is not present in the crystal structure of mGluR1 dimer (4OR2) (Wu et al., 2014), conformational changes of the dimerized, isolated ECDs upon agonist binding have been observed in previously reported crystal structures (Kunishima et al., 2000). Such a conformational change of the dimerized ECD is likely to induce a conformational change in the linker regions. Since the dimer interface observed in the crystal structure of mGluR1 (4OR2) is formed between the two TM1 helices to which the linker peptides are attached, the putative ligand-induced conformational changes of the linker peptides are unlikely to directly affect the remaining TM helices. Therefore, an energy coupling between ligand binding to the ECD region and reorganization of the 7 helices in the transmembrane domain is difficult to be envisioned. We argue that the first mechanism is simply not to apply to class-C GPCRs. Thus, if the second mechanism is more likely to be the case, what exactly constitutes the neo-ligand?

The crystal structure of mGluR1 (4OR2) indicates that the ECL2 of each 7-TM domains forms a β-hairpin that protrudes into the solvent and is located right above the NAM binding pocket. Interactions between the linker peptide and the β-hairpin include hydrophobic interactions, a salt-bridged bond between the linker Arg584 and the ECL2 Glu741, as well as a few main-chain hydrogen bonds forming a small β-sheet that expands from the β-hairpin of ECL2. Although β-hairpin structures have been reported in the ECL2 structures of some class-A GPCRs [e.g. CXCR4 and δ-opioid receptor (Wu et al., 2010; Fenalti et al., 2014)], the type of interaction between an N-terminal peptide and an ECL2 β-hairpin that is observed in mGluR1 crystal structure has not been found in structures of other GPCRs. In contrast, among class-C GPCRs both the linker peptides and the ECL2 regions are conserved in lengths, respectively. Therefore, the linker-ECL2 interaction that is observed in mGluR1 structure may be conserved in other class-C GPCRs. One may speculate that the ligand-binding signal would disrupt the linker-ECL2 interactions, thus freeing the β-hairpin to interact with a one or more new partners. Upon release, the β-hairpin may serve as a self-tethered ligand that has the ability to trigger the activation of the 7-TM domain (Fig. 1). A similar activation mechanism was experimentally verified in the class-A GPCR, PAR1 (protease activated receptor-1), where the new N-terminal end of the peptide, generated by thrombin cleavage, serves as an agonist to activate the PAR1 7-TM domain (Trejo et al., 1996). An alternative possibility is that the linker peptide released from the β-hairpin serves as a trigger for the activation of the 7-TM domain. A conceptually similar mechanism was proposed for class-C GPCRs as the “peptide-linker model” of activation (Margeta-Mitrovic et al., 2001), prior to the availability of structural information for dimerization. However, based on the mGluR1 crystal structure and considering the restriction from the dimerized ECDs, it seems easier for the ECL2 β-hairpin rather than the linker peptide to serve as the trigger fitting into the ligand-binding pocket.

**Figure 1:**
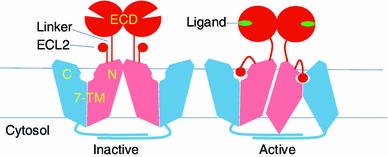
Schematic diagram of a hypothetical mechanism for the activation of a class-C GPCR dimer

Why then does class-C GPCRs require dimerization in signaling? The subunits of the dimer provide a point of physical reference relative to each other. First, ECD rearrangement upon binding of orthosteric agonists is translated into a relative movement between the two linker peptides. Secondly, because of the dimerization, the movement of the linker peptides can be translated into a relative movement between the linker peptide and the β-hairpin in each subunit, thus releasing of the β-hairpin from the linker peptide. In contrast, without formation of dimers, the information of the conformational change of ECD would not result in the release of the β-hairpin. Data from previous reports on class-C GPCR activation are consistent with such an interpretation. For example, GBR is a heterodimer, and its two subunits GB1 and GB2 bear functions complementary to each other in signal transmission. In particular, the GB1 subunit binds to orthosteric agonists at its ECD but contains a 7-TM domain unable to activate downstream G-proteins. In contrast, the GB2 subunit can activate downstream G-proteins at its cytosolic side of the 7-TM domain but contains an ECD unable to bind orthosteric ligands. However, it has been shown that switching the ECDs of GB1 and GB2 subunits has no effects on either levels or duration of activation of the GBR heterodimer (Margeta-Mitrovic et al., 2001; Galvez et al., 2001; Havlickova et al., 2002). To interpret this result, one should note that most of the linker regions from these two subunits are conserved in terms of amino acid types (TLVIKTFRFLS-QKFL in GB1 vs. TIILEQLRKIS-LPLY in GB2, with breaking points indicated by hyphens). Thus, the interactions between the linker peptide and β-hairpin in each subunit of the chimeric dimer may remain the same as the native dimer, especially if the interactions mainly consist of main-chain hydrogen-bonds. In addition, replacing the ECD of GB2 with that of GB1 creates a chimeric subunit termed GB1/2, and a homodimer formed by GB1/2 displayed wild type-like activity. Importantly, replacing the GB2 linker peptide with a highly flexible poly-Gly peptide abolished the signaling, while the same mutation in GB1 showed no effect. In fact, as long as the linker peptide of GB2 is preserved, the activity of the dimer is maintained in a variety of chimeric combinations (Margeta-Mitrovic et al., 2001). These observations suggest that the linker peptide in GB2, which contains a functional 7-TM domain, is more important than that in GB1, whose 7-TM domain is non-functional in terms of activating downstream G-proteins. Moreover, point mutations in the linker region of the CaS homodimer, in which both subunits contain a functional 7-TM domain, may result in the loss of signal transduction (Ray et al., 2007). All these data suggest that the relative movement, i.e. the change in distance between the two linker regions, is essential for triggering the activation of the 7-TM domain, and support the importance of the linker-ECL2 interaction. It is worthy to mention that such a mechanism of dimerization-dependent activation found in class-C GPCRs is likely to be fundamentally different from potential mechanisms involving oligomerization seen in other GPCRs that do not have the linker peptide.

In homodimers of class-C GPCRs, only one subunit can be activated stochastically, which is similar to what has been observed for the heterodimer of GBR. For instance, it has been shown for mGluRs that activation of one subunit inhibits the other in a process that appears to be random (Goudet et al., 2005; Hlavackova et al., 2005). This phenomenon is likely to be caused by the close contact between the two subunits, enforced by interactions between their respective extracellular, 7-TM, and cytosolic regions (Kniazeff et al., 2011). Let’s assume that the activation progress of the 7-TM domain of a subunit in a class-C GPCR dimer is similar to that of the class-A GPCRs (Rasmussen et al., 2011). The activation of one subunit in the dimer, i.e. being enabled to interact with the downstream G-protein, would result in a large conformational change, particularly expanding the cytosolic side of the 7-TM domain. It appears that one such a large conformational change can be accommodated in the closely packed class-C GPCR dimer, but the simultaneous change of the two of such activated 7-TM domains might be prohibited (Fig. 1).

Future studies on the full-length structures of class-C GPCRs, as well as more detailed mutation analysis on the linker-ECL2 interaction will be necessary to verify the current hypothesis on the mechanisms for the activation of class-C GPCRs and in particular the mechanisms for signal transduction from the ECD dimer to the 7-TM dimer.

## Footnotes

This work was supported by the National Basic Research Program (973 Program) (No. 2011CB910301 to XCZ).

Xuejun C. Zhang, Jianfeng Liu, and Daohua Jiang declare that they have no conflict of interest. This article does not contain any studies with human or animal subjects performed by the any of the authors.
